# Galanin Receptor 2 Is Involved in Galanin-Induced Analgesic Effect by Activating PKC and CaMKII in the Nucleus Accumbens of Inflammatory Pain Rats

**DOI:** 10.3389/fnins.2020.593331

**Published:** 2021-01-21

**Authors:** Mengnan Li, Xiaomin Zhang, Chongyang Li, Yanan Liu, Shuang Yang, Shilian Xu

**Affiliations:** ^1^Department of Physiology, School of Basic Medicine, Kunming Medical University, Kunming, China; ^2^Department of Oncology, Affiliated Hospital, Yunnan University, Kunming, China

**Keywords:** galanin receptor 2, inflammatory pain, analgesic effect, nucleus accumbens, CaMKII, PKC

## Abstract

It has been reported that galanin has an analgesic effect via activating galanin receptors (GALRs). This study focused on the involvement of GALR2 in the galanin-induced analgesic effect and its signaling mechanism in the nucleus accumbens (NAc) of inflammatory rats. Animal models were established through injecting carrageenan into the plantar of rats’ left hind paw. The results showed that GALR2 antagonist M871 weakened partially the galanin-induced increases in hind paw withdrawal latency (HWL) to thermal stimulation and hind paw withdrawal threshold (HWT) to mechanical stimulation in NAc of inflammatory rats. Moreover, the GALR2 agonist M1145 prolonged the HWL and HWT, while M871 blocked the M1145-induced increases in HWL and HWT. Western blotting showed that the phosphorylation of calcium/calmodulin-dependent protein kinase II (p-CaMKII) and protein kinase C (p-PKC) in NAc were upregulated after carrageenan injection, while p-PKC and p-CaMKII were downregulated after intra-NAc administration of M871. Furthermore, the CaMKII inhibitor KN93 and PKC inhibitor GO6983 attenuated M1145-induced increases in HWL and HWT in NAc of rats with inflammatory pain. These results prove that GALR2 is involved in the galanin-induced analgesic effect by activating CaMKII and PKC in NAc of inflammatory pain rats, implying that GALR2 agonists probably are potent therapeutic options for inflammatory pain.

## Introduction

Pain is a dominating feature of inflammation, but the underlying mechanisms of inflammatory pain resolution are not fully understood. Thus, the treatment of inflammatory pain has always been a major issue in the clinic. Non-steroidal anti-inflammatory drugs are extensively used in the treatment of inflammatory pain associated with a number of diseases. However, there are safety concerns regarding the use of these drugs, such as clinically relevant gastrointestinal, cardiovascular, and renal damage. Therefore, it is imperative to develop new drugs and tools that do not induce significant side effects.

In addition to endogenous opioids, which are important neuropeptides for the modulation of pain, other neuropeptides, such as substance P, are also involved in neurogenic inflammation (O’Connor et al., 2004). Galanin is an important neuropeptide that is widely distributed peptide in the peripheral tissues and central nervous system (CNS), participates in the regulation of nociceptive information (Amorim et al., 2015; Li et al., 2017). A study found that the hind paw withdrawal latencies (HWLs) were increased after intra-anterior cingulate cortex (ACC) administration of galanin in rats (Zhang et al., 2017b). Our studies also showed that intra-nucleus accumbens (NAc) administration of galanin increased the HWLs in rats (Xu S.L. et al., 2012; Yang et al., 2015; Zhang et al., 2019). Galanin plays a role by activating its specific receptors. Galanin receptors (GALRs), including GALR1-3, have been identified (Webling et al., 2012). It has been demonstrated that galantide, a non-selective GALRs antagonist, attenuates galanin-induced analgesic effect in rats (Xu S.L. et al., 2012; Yang et al., 2015). In this study, the GALR2-specific agonist M1145 (Runesson et al., 2009; Saar et al., 2013) and the GALR2-specific antagonist M871 (Sollenberg et al., 2006) were used to investigate the analgesic effect of GALR2 in rats with inflammatory pain. We hope to provide a new insight into the treatment of inflammatory pain.

The signaling pathways of the three GALRs are essentially different (Lang et al., 2015), and the signaling transduction mechanism of the analgesic effects of galanin and its receptors on inflammatory pain is not fully illustrated yet. Calcium/calmodulin-dependent protein kinase II (CaMKII) and protein kinase C (PKC) belong to a family of serine/threonine kinases that are all phospholipase C (PLC)-dependent Ca^2+^-related protein kinases. Activation of multiple PKC isoforms is important for the development of central and/or peripheral sensitization in persistent pain conditions (He and Wang, 2019). Besides, CaMKII also plays important roles in pain control (Crown et al., 2012; Kadic et al., 2014; He et al., 2016; Yao et al., 2016). N-methyl-D-aspartate (NMDA) receptors contribute to the up-regulation of inflammatory pain sensitization during inflammation (Tan et al., 2010), whereas KN93 (CaMKII inhibitor) and chelerythrine (PKC inhibitor) can decrease hyperalgesia and allodynia by regulating the phosphorylation of NMDA receptor subunits (Liu et al., 2014; Bu et al., 2015).

The NAc is an important central nucleus for pain modulation, which consists two regions: the core and shell (Baliki et al., 2013; Duan et al., 2015; Salgado and Kaplitt, 2015; Watanabe et al., 2018; Zhang et al., 2019). Our previous study showed that galanin in NAc had an analgesic effect and that this effect was blocked by non-specific GALRs antagonist galantide in rats with inflammatory pain (Yang et al., 2015). In this work we explored whether the CaMKII and/or PKC signaling pathways were implicated in the analgesic effect of GALR2 in NAc of inflammatory rats. Our results will improve the understanding of the molecular mechanism of galanin-induced analgesic effect.

## Materials and Methods

### Animals

Male Sprague-Dawley rats weighing 180–250 g were supplied by the Experimental Animal Center of Kunming Medical University (Kunming, Yunnan, China). The rats were kept in cages with free access to food and water. The room temperature was kept at 22 ± 1°C, and the animals were housed under strictly controlled lighting conditions. All experimental protocols were tested in accordance with the approval of the “Animal Care and Use Committee at Kunming Medical University” and “National Institute of Health Guide for the Care and Use of Laboratory Animals.”

### Chemicals

Solutions for intra-NAc administration containing either 2 nmol rat galanin (Tocris, Bristol, United Kingdom); 0.1, 1, or 2 nmol M1145 (Tocris, Bristol, United Kingdom) were prepared in 1 μl of 0.9% sterilized saline; solutions containing either 6, 12, or 24 μg of KN93 (C_26_H_29_ClN_2_O_4_S; EMD Biosciences, Inc., La Jolla, CA, United States); or 12, 24, or 36 μg of GO6983 (C_26_H_26_N_4_O_3_; MCE, United Kingdom) were prepared in 1 μl of 1% dimethyl sulfoxide (DMSO). M871 (Tocris, Bristol, United Kingdom) was dissolved in 1 μl of 6% acetonitrile at a concentration of 2 nmol for intra-NAc administration. 2 mg of carrageenan (Sigma-Aldrich, St. Louis, MO, United States) was dissolved in 0.1 ml sterilized saline, and was injected into the plantar of the left hind paw.

### Antibodies

Table 1 shows a list of all antibodies used.

**TABLE 1:** A list of antibodies.

Antigen	Description of immunogen	Source, host species, catalog No., RRID	Concentration used
GAPDH	Monoclonal antibody is produced by immunzing animals with a synthetic peptide corresponding to residues near the carboxy terminus of human GAPDH	Cell Signaling Technology, Rabbit monoclonal Cat# 5174, RRID:AB_10622025	1:1000 (WB)
CaMKII (pan)	CaMKII (pan) (D11A10) Rabbit mAb detects endogenous levels of total CaMKII protein. The peptide sequence used as the antigen is 100% conserved between CaMKII-alpha, gamma and delta, and 88% conserved in CaMKII-beta. Synthetic peptide surrounding Val184 of human CaMKII-alpha	Cell Signaling Technology, Rabbit monoclonal Cat# 4436, RRID:AB_10545451	1:1000 (WB)
CaMKII (Thr286) Phosphate	recognizes endogenous levels of CamKII-α protein only when phosphorylated at Thr286. This antibody also recognizes endogenous levels of CamKII-β and CamKII-γ protein only when phosphorylated at Thr287 synthetic phosphopeptide corresponding to residues surrounding Thr287 of human CamKII-β protein	Cell Signaling Technology, Rabbit monoclonal Cat# 12716, RRID:AB_2713889	1:1000 (WB)
PKC alpha [Y124]	Synthetic peptide within Human PKC alpha aa 650 to the C-terminus. The exact sequence is proprietary	Abcam, Rabbit monoclonal Ab32376, RRID:AB_777294	1:5000 (WB)
PKC alpha (phospho T497)	Synthetic peptide (the amino acid sequence is considered to be commercially sensitive) within Human PKC alpha (phospho T497). The exact sequence is proprietary	Abcam, Rabbit monoclonal Ab76016, RRID:AB_1310584	1:5000 (WB)

### Behavioral Tests

The HWL to thermal stimulation and the hind paw withdrawal threshold (HWT) to mechanical stimulation were measured as previously described (Sun et al., 2003; Duan et al., 2015; Yang et al., 2015). The HWL to thermal stimulation was assessed by a Hot-Plate (YLS-6B, China), with the temperature kept at 52 ± 0.2°C. The unilateral hind paw was put on the hot-plate gently and ensured that the entire ventral surface of the hind paw touched the hot-plate. The time to hind paw withdrawal was measured in seconds (s) and recorded as the HWL to thermal stimulation. The HWT to mechanical stimulation was determined by a Randall-Selitto meter (Ugo Basile, 37215, Italy), that a wedge-shaped pusher with a loading rate of 30 g/s was applied to the dorsal surface of the rat’s hind paw and the magnitude of the mechanical stimulation required to initiate the struggle response was measured as HWT.

Before the experiment, all rats were acclimated to behavioral tests for 4–5 days, the HWL to thermal stimulation was normally maintained between 3 and 6 s, and the HWT to mechanical stimulation was maintained between 4 and 7 g. When measuring HWL or HWT, if the rat did not withdrawal the hind paw after 15 s (or over 15 g), the paw would be lifted by the trier to avoid tissue damage.

### Intra-NAc Catheter Implantation

Rats were first anesthetized by intraperitoneal injection of sodium pentobarbital (50 mg/kg) and placed on a stereotaxic instrument. Then a stainless-steel catheter with an outer diameter of 0.8 mm was implanted into the NAc (Bregma: +1.7 mm; Left or right of the midline: 1.6 mm; Ventrally to the surface of skull: 7.0 mm) (Paxinos and Watson, 1998) and was secured to the skull with dental acrylic. Rats were then allowed to recover for 2–3 days.

### Carrageenan-Induced Inflammatory Pain Model

On the day of the experiment, an inflammatory pain model was established through subcutaneous injection of 0.1 ml of 2% carrageenan into the plantar of the left hind paw of rat (Sun et al., 2003; Xiong et al., 2005; Yang et al., 2015; Zhang et al., 2017a). The contralateral paw was untreated. Then, each animal model of inflammatory pain received an intra-NAc injection of the drugs.

### Intra-NAc Injection

Three hours after carrageenan injection, each HWL and HWT were measured three times which were averaged to obtain a mean value as the baseline HWL and HWT. Each HWL or HWT test should be 5 min apart from the last test to prevent discomfort or injury.

Then, a stainless-steel needle with an outer diameter of 0.4 mm was inserted into the stainless-steel catheter and its tip exceeded the stainless-steel catheter by 1 mm for bilateral intra-NAc injection. The HWL and HWT were measured 5, 10, 15, 20, 30, 45, and 60 min after bilateral intra-NAc injection, and each HWL or HWT was expressed as percentage changes from the baseline HWL. The formula is as follows:

$$\text{HWL}\%=\frac{\begin{array}{c} \mathrm{HWL}\,\mathrm{measured}\,\mathrm{after}\,\mathrm{intra}\,\text{-}\,\mathrm{NAc}\,\mathrm{injection}\\ -\mathrm{baseline}\,\mathrm{HWL} \end{array}}{\mathrm{baseline}\,\mathrm{HWL}}\times 100\%$$

The drugs were injected into the core of NAc, and the location of the needle tip was verified at the end of each experiment. Only data from the animals with tip of the needle located in the NAc were used for statistical analysis.

### Western Blot

Rats were deeply anesthetized with 4% isoflurane and euthanized, and tissue of bilateral NAc area was collected. The Western blot assay was operated as previously described (Duan et al., 2015; Yang et al., 2015). The expression of total-PKC (t-PKC) alpha, phospho-PKC (p-PKC) alpha, total-CaMKII (t-CaMKII) alpha and phospho-CaMKII (p-CaMKII) alpha were measured, and the level of glyceraldehyde-3-phosphate dehydrogenase (GAPDH) was measured as internal control. The relative band density was assessed by ImageJ software. Western blotting of the protein samples was repeated at least three times.

### Statistical Analysis

The data were analyzed using GraphPad Prism 5 software, and presented as the mean ± SEM. Western blot results were assessed by one-way ANOVA followed by Tukey’s multiple comparison test, behavioral tests were analyzed by two-way repeated-measures ANOVA followed by Bonferroni *post hoc* test, and *P* < 0.05 was considered as statistically significant.

## Results

### The GALR2 Antagonist M871 Attenuated the Galanin-Induced Analgesic Effect in NAc of Inflammatory Pain Rats

**FIGURE 1:**
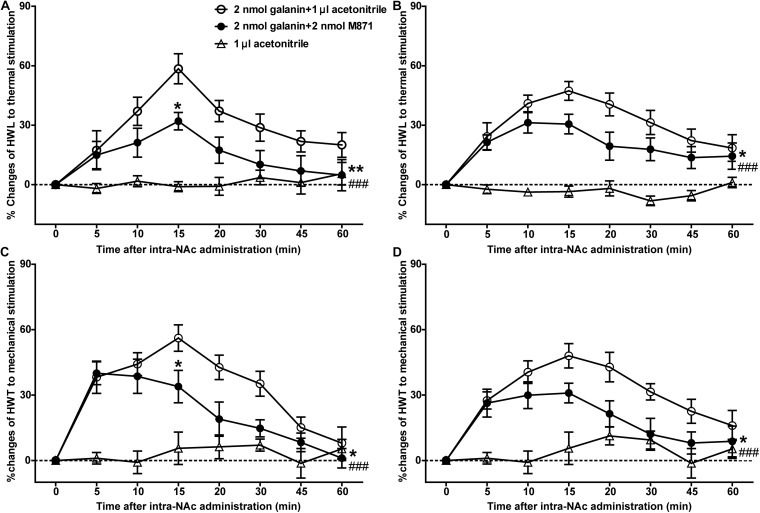
Effect of M871 on galanin-induced increases in HWL and HWT in NAc of inflammatory pain rats **(A,C)**: left hind paw; **(B,D)**: right hind paw. 2 nmol galanin was bilaterally injected into NAc at 0 min, and 2 nmol M871 or 1 μl of acetonitrile as a control was bilaterally injected into NAc at 5 min. *Represents the difference between the group treated with galanin + M871 and the group treated with galanin + acetonitrile; #Represents the difference between the group treated with galanin + M871 and the group treated with acetonitrile alone. *P < 0.05, **P < 0.01, compared the galanin + M871-treated group with the galanin + acetonitrile-treated group. ^###^*P* < 0.001 compared galanin + M871-treated group with the acetonitrile alone group.

Our previous study showed that galanin had an analgesic effect in NAc of inflammatory pain rats and that the expression of GALR2 was upregulated in NAc 3 h after injection of carrageenan (Yang et al., 2015). To explore whether this analgesic effect was mediated by GALR2, 3 h after carrageenan injection, two groups of inflammatory pain rats received an intra-NAc injection of 2 nmol galanin followed by an intra-NAc injection of 2 nmol GALR2 antagonist M871 (*n* = 8) or 1 μl of 6% acetonitrile as a control (*n* = 9) 5 min later. Compared to the group treated with galanin + acetonitrile, galanin-induced increases in HWL to thermal stimulation (Left hind paw: *F*_(__1_,_75__)_ = 9.48, *P* = 0.0076; Right hind paw: *F*_(__1_,_75__)_ = 5.246, *P* = 0.0369) and HWT to mechanical stimulation [Left hind paw: *F*_(__1_,_75__)_ = 4.551, *P* = 0.0498; Right hind paw: *F*_(__1_,_75__)_ = 6.366, *P* = 0.0234] were effectively blocked by the intra-NAc injection of GALR2 antagonist M871. And when comparing the HWL and HWT of the two groups at each time point, there was a significant difference in the left HWL and HWT after 15 min of galanin injection (Figure 1), which suggested that the analgesic effect of galanin on inflammatory pain might be mediated through the GALR2 activation in NAc of rats. The significance of the difference between the groups was determined by two-way ANOVA for repeated measurements followed by Bonferroni *post hoc* test.

In order to further study the blocking effect of M871 on galanin induced analgesia, inflammatory pain rats were given an intra-NAc injection of 6% acetonitrile (*n* = 8) alone. Compared to the group treated with acetonitrile alone, the HWL [Left hind paw: *F*_(__1_,_70__)_ = 42.05, *P* < 0.0001; Right hind paw: *F*_(__1_,_70__)_ = 29.82, *P* < 0.0001] and HWT [Left hind paw: *F*_(__1_,_70__)_ = 22.15, *P* = 0.0003; Right hind paw: *F*_(__1_,_70__)_ = 43.43, *P* < 0.0001] were increased in the group treated with galanin + M871, suggesting the antinociception of galanin was blocked partially by GALR2 antagonist M871 (Figure 1). This result implied that galanin in NAc had an analgesic effect on inflammatory pain by activating other GALRs.

### Intra-NAc Injection of GALR2 Agonist M1145 Increased the HWL and HWT in Inflammatory Pain Rats

**FIGURE 2:**
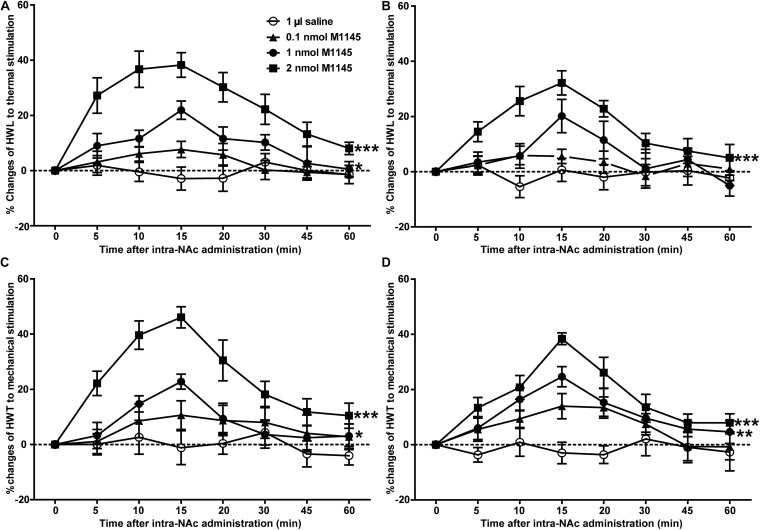
Effect of M1145 on the HWL and HWT in NAc of inflammatory pain rats **(A,C)**: left hind paw; **(B,D)**: right hind paw. 0.1, 1, 2 nmol M1145 or 1 μl of saline as a control was bilaterally injected into NAc respectively at 0 min. The data are presented as the mean ± SEM. *P < 0.05, **P < 0.01, ***P < 0.001 compared with the saline group.

To investigate the analgesic effect of GALR2 activation on inflammatory pain, four groups of carrageenan-treated rats received an intra-NAc administration of 1 μl of either 0.1 nmol (*n* = 7), 1 nmol (*n* = 9), 2 nmol (*n* = 7) GALR2 agonist M1145 or 0.9% saline (*n* = 7) 3 h after carrageenan injection. Compared with those of saline group, the HWL to thermal stimulation significantly prolonged after intra-NAc administration of 1 nmol [Left hind paw: *F*_(__1_,_84__)_ = 6.94, *P* = 0.0196; Right hind paw: *F*_(__1_,_84__)_ = 2.02, *P* = 0.1796] and 2 nmol [Left hind paw: *F*_(__1_,_72__)_ = 85.63, *P* < 0.0001; Right hind paw: *F*_(__1_,_72__)_ = 46.59, *P* < 0.0001] M1145, but for rats received 0.1 nmol M1145, the HWL was not significantly increased [Left hind paw: *F*_(__1_,_72__)_ = 2.15, *P* = 0.1683; Right hind paw: *F*_(__1_,_72__)_ = 1.38, *P* = 0.2636] (Figures 2A,B). While the HWT to mechanical stimulation of rats treated with 1 nmol [Left hind paw: *F*_(__1_,_84__)_ = 5.32, *P* = 0.0369; Right hind paw: *F*_(__1_,_84__)_ = 11.31, *P* = 0.0046] and 2 nmol [Left hind paw: *F*_(__1_,_72__)_ = 37.74, *P* < 0.0001; Right hind paw: *F*_(__1_,_72__)_ = 31.85, *P* = 0.0002] M1145 significantly prolonged, but not with 0.1 nmol M1145 [Left hind paw: *F*_(__1_,_72__)_ = 1.80, *P* = 0.2044; Right hind paw: *F*_(__1_,_72__)_ = 4.46, *P* = 0.0564] (Figures 2C,D). The data were analyzed by two-way ANOVA (repeated-measures). The results confirmed that GALR2 activation had an antinociceptive effect in the NAc of rats with inflammatory pain.

### The GALR2 Antagonist M871 Reversed the M1145-Induced Increases in HWL and HWT in NAc of Inflammatory Pain Rats

**FIGURE 3:**
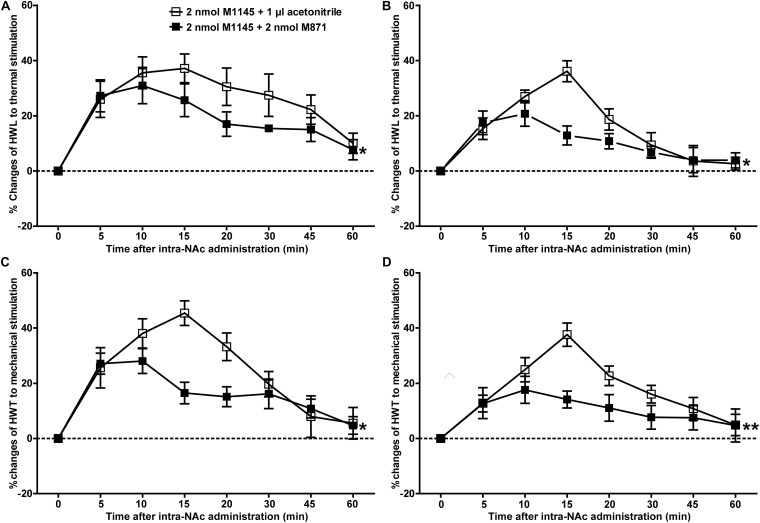
Effect of M871 on M1145-induced increases in the HWL and HWT of inflammatory pain rats **(A,C)**: left hind paw; **(B,D)**: right hind paw. 2 nmol M1145 was bilaterally injected into NAc at 0 min, and 2 nmol M871 or 1 μl of acetonitrile as a control was bilaterally injected into NAc at 5 min. The data are presented as the mean ± SEM. *P < 0.05, **P < 0.01 compared with the acetonitrile group.

To further confirm the analgesic effect of GALR2 activation in the NAc on inflammatory pain, carrageenan-treated rats received an intra-NAc administration of 2 nmol GALR2 agonist M1145, and 5 min later, rats received an intra-NAc injection of 2 nmol GALR2 antagonist M871 (*n* = 8) or 1 μl of 6% acetonitrile as a control (*n* = 8). Compared with those of M1145 + acetonitrile group, GALR2 antagonist M871 attenuated GALR2 agonist M1145-induced increases in the HWL to thermal stimulation [Left hind paw: *F*_(__1_,_70__)_ = 5.64, *P* = 0.0324; Right hind paw: *F*_(__1_,_70__)_ = 5.16, *P* = 0.0394] (Figures 3A,B) and HWT to mechanical stimulation [Left hind paw: *F*_(__1_,_70__)_ = 6.00, *P* = 0.028; Right hind paw: *F*_(__1_,_70__)_ = 9.10, *P* = 0.0092] (Figures 3C,D). The data were analyzed by two-way repeated-measures ANOVA. This result further implied that GALR2 activation had an analgesic effect on inflammatory pain in NAc of rats.

### The Expressions of p-PKC and p-CaMKII Were Upregulated in NAc of Inflammatory Pain Rats

**FIGURE 4:**
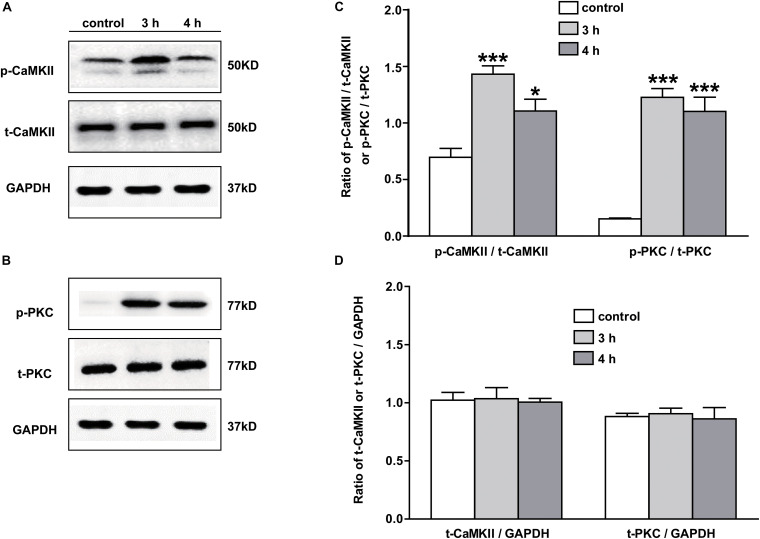
The expression of PKC and CaMKII in NAc of inflammatory pain rats 3 h: 3 h after carrageenan injection; 4 h: 4 h after carrageenan injection. **(A,B)** Representative Western blots of p-CaMKII, t-CaMKII, p-PKC, t-PKC and GAPDH. **(C)** Histograms showing the p-CaMKII/t-CaMKII ratio and p-PKC/t-PKC ratio. **(D)** Histograms showing the t-CaMKII/GAPDH ratio and t-PKC/GAPDH ratio. The data are presented as the mean ± SEM. *P < 0.05, ***P < 0.001 compared with the saline group.

The signaling mechanism underlying the analgesic effect of galanin in NAc of inflammatory pain rats is unclear. In this study, the expressions of PKC and CaMKII were measured by Western blotting. The results showed that p-CaMKII in NAc was significantly upregulated 3 (*n* = 4, *q* = 8.44, *P* < 0.001) and 4 h (*n* = 4, *q* = 4.71, *P* < 0.05) after carrageenan injection, as shown in Figures 4A,C. Meanwhile, the p-PKC also significantly increased in NAc 3 (*n* = 4, *q* = 12.55, *P* < 0.001) and 4 h (*n* = 4, *q* = 11.08, *P* < 0.001) after carrageenan injection (Figures 4B,C). But there were no significantly changes in the levels of t-CaMKII (Figures 4A,D) and t-PKC (Figures 4B,D) either 3 (t-CaMKII: *n* = 4, *q* = 0.18, *P* > 0.05; t-PKC: *n* = 4, *q* = 0.39, *P* > 0.05) or 4 h (t-CaMKII: *n* = 4, *q* = 0.23, *P* > 0.05; t-PKC: *n* = 4, *q* = 0.32, *P* > 0.05) after carrageenan injection, which suggested that the PKC and CaMKII signaling pathways in NAc might be involved in inflammatory pain in rats. The differences were analyzed by one-way ANOVA followed by Tukey’s multiple comparisons test.

### The GALR2 Antagonist M871 Downregulated p-PKC and p-CaMKII Expressions in NAc of Inflammatory Pain Rats

**FIGURE 5:**
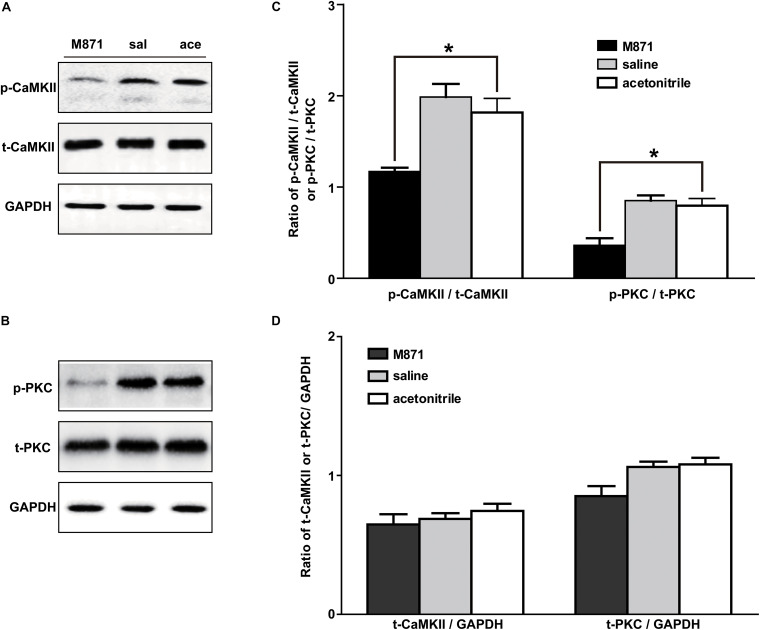
Effect of M871 on PKC or CaMKII expression in NAc of inflammatory pain rats sal: saline; ace: acetonitrile. **(A,B)** Representative Western blots of p-CaMKII, t-CaMKII, p-PKC, t-PKC and GAPDH. **(C)** Histograms showing the p-CaMKII/t-CaMKII ratio and p-PKC/t-PKC ratio. **(D)** Histograms showing the t-CaMKII/GAPDH ratio and t-PKC/GAPDH ratio. The data are presented as the mean ± SEM. *P < 0.05 compared with the acetonitrile group.

We next investigated whether the PKC and/or CaMKII signaling pathways in NAc of inflammatory pain rats were mediated by GALR2 activation. Three hours after carrageenan injection, 2 nmol GALR2 antagonist M871, 1 μl of normal saline or 1 μl of 6% acetonitrile was injected into the NAc. Fifteen minutes after injection, proteins in bilateral NAc tissues were rapidly extracted, and the levels of PKC and CaMKII were determined by Western blotting. As shown in Figure 5, the intra-NAc administration of GALR2 antagonist M871 significantly downregulated the expressions of p-CaMKII (*n* = 3, *q* = 5.20, *P* < 0.05) and p-PKC (*n* = 3, *q* = 6.09, *P* < 0.05) compared with the acetonitrile-treated group, but the levels of t-PKC (*n* = 3, *q* = 4.21, *P* > 0.05) and t-CaMKII (*n* = 3, *q* = 1.70, *P* > 0.05) were not significantly different (Figure 5). The results were analyzed by one-way ANOVA followed by Tukey’s multiple comparisons test.

These results showed that GALR2 antagonist M871 blocked GALR2 and then downregulated p-PKC and p-CaMKII expressions, suggesting that GALR2 activation exerted an analgesic effect through activating PKC and CaMKII in NAc of inflammatory pain rats.

### The CaMKII Inhibitor KN93 and the PKC Inhibitor GO6983 Attenuated the Analgesic Effect of M1145 in NAc of Inflammatory Pain Rats

**FIGURE 6:**
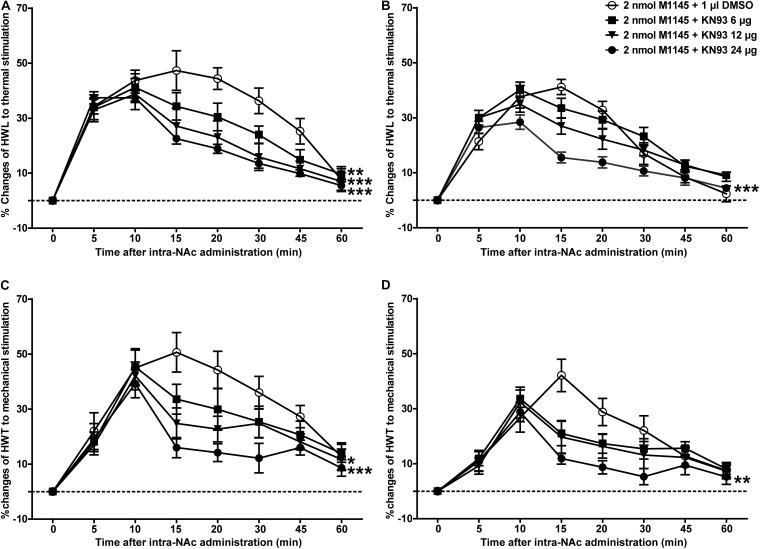
Effect of KN93 on the M1145-induced increases in HWL and HWT in NAc of inflammatory pain rats **(A,C)** left hind paw; **(B,D)** right hind paw. 2 nmol M1145 was bilaterally injected into NAc at 0 min, and 6, 12, 24 μg KN93 or 1 μl of DMSO as a control was bilaterally injected into NAc respectively at 5 min. The data are presented as the mean ± SEM. *P < 0.05, **P < 0.01, ***P < 0.001 compared with the DMSO control group.

To further determine whether CaMKII is involved in GALR2-mediated analgesic effects, 3 h after carrageenan injection, 2 nmol GALR2 agonist M1145 was injected into the NAc to activate GALR2, and 5 min after intra-NAc injection of M1145, 24 μg (*n* = 12), 12 μg (*n* = 8), 6 μg (*n* = 8) of the CaMKII inhibitor KN93 or 1% DMSO as a control (*n* = 12) was injected into the NAc respectively. Compared with that in the DMSO-treated group, the M1145-induced increase in HWL to thermal stimulation was attenuated in a dose-dependent manner by the intra-NAc administration of 24 μg [Left hind paw: *F*_(__1_,_110__)_ = 40.05, *P* < 0.0001; Right hind paw: *F*_(__1_,_110__)_ = 19.36, *P* = 0.0002], 12 μg [Left hind paw: *F*_(__1_,_90__)_ = 16.43, *P* = 0.0007; Right hind paw: *F*_(__1_,_90__)_ = 2.78, *P* = 0.1486] of CaMKII inhibitor KN93, but for rats received 6 μg of KN93, only left HWL was significantly increased [Left hind paw: *F*_(__1_,_90__)_ = 11.39, *P* = 0.0034; Right hind paw: *F*_(__1_,_90__)_ = 0.07, *P* = 0.7990] (Figures 6A,B). And the HWT to mechanical stimulation was also significantly attenuated by the intra-NAc administration of 24 μg [Left hind paw: *F*_(__1_,_110__)_ = 17.78, *P* = 0.0004; Right hind paw: *F*_(__1_,_110__)_ = 8.58, *P* = 0.0078], 12 μg [Left hind paw: *F*_(__1_,_90__)_ = 5.00, *P* = 0.0383; Right hind paw: *F*_(__1_,_90__)_ = 1.53, *P* = 0.2322] of KN93, but not 6 μg of KN93 [Left hind paw: *F*_(__1_,_90__)_ = 1.39, *P* = 0.2541; Right hind paw: *F*_(__1_,_90__)_ = 0.81, *P* = 0.379]. As shown in Figures 6C,D, the differences were analyzed by two-way repeated-measures ANOVA.

**FIGURE 7:**
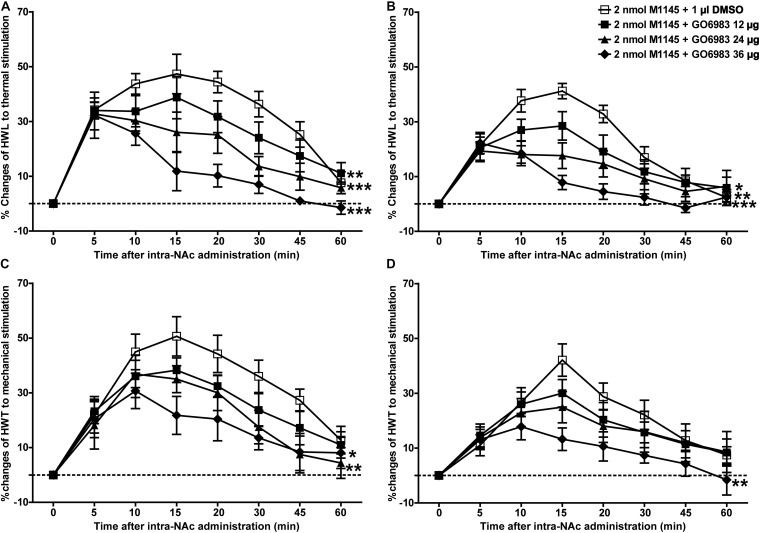
Effect of GO6983 on the M1145-induced increases in HWL and HWT in NAc of inflammatory pain rats **(A,C)** left hind paw; **(B,D)** right hind paw. 2 nmol M1145 was bilaterally injected into NAc at 0 min, and 12, 24, 36 μg GO6983 or 1 μl of DMSO as a control was bilaterally injected into NAc respectively at 5 min. The data are presented as the mean ± SEM. *P < 0.05, **P < 0.01, ***P < 0.001 compared with the DMSO control group.

Meanwhile, to further study whether PKC is a downstream signaling molecule associated with the analgesic effect of GALR2 activation on inflammatory pain, 2 nmol GALR2 agonist M1145 was injected into the NAc of rats with inflammatory pain, and 5 min later, 12 μg (*n* = 8), 24 μg (*n* = 8), 36 μg (*n* = 8) of the PKC inhibitor GO6983 or 1% DMSO as a control (*n* = 12) was injected into the NAc, respectively. Compared with that in the DMSO-treated group, the M1145-induced increase in HWL to thermal stimulation was attenuated by the intra-NAc administration of 36 μg [Left hind paw: *F*_(__1_,_90__)_ = 63.64, *P* < 0.0001; Right hind paw: *F*_(__1_,_90__)_ = 46.21, *P* < 0.0001], 24 μg [Left hind paw: *F*_(__1_,_90__)_ = 33.71, *P* < 0.0001; Right hind paw: *F*_(__1_,_90__)_ = 13.24, *P* = 0.0019] or 12 μg [Left hind paw: *F*_(__1_,_90__)_ = 10.00, *P* = 0.0054; Right hind paw: *F*_(__1_,_90__)_ = 4.84, *P* = 0.0411] of PKC inhibitor GO6983 (Figures 7A,B). The M1145-induced increase in HWT to mechanical stimulation was also attenuated by the intra-NAc administration of 36 μg [Left hind paw: *F*_(__1_,_90__)_ = 14.42, *P* = 0.0013; Right hind paw: *F*_(__1_,_90__)_ = 10.26, *P* = 0.0049], 24 μg [Left hind paw: *F*_(__1_,_90__)_ = 7.77, *P* = 0.0122; Right hind paw: *F*_(__1_,_90__)_ = 1.22, *P* = 0.2833], but not 12 μg [Left hind paw: *F*_(__1_,_90__)_ = 3.53, *P* = 0.0764; Right hind paw: *F*_(__1_,_90__)_ = 0.62, *P* = 0.4418] of PKC inhibitor GO6983 (Figures 7C,D). The differences were analyzed by two-way repeated-measures ANOVA.

These results again proved that both PKC and CaMKII were involved in the analgesic effect of GALR2 in NAc of inflammatory pain rats.

## Discussion

The role of neuropeptide galanin in pain modulation has been well-established, and it has been suggested that galanin may be a potential target for novel therapies. Some studies have recently proven that galanin has an analgesic effect on inflammatory pain (Yang et al., 2015; Zhang et al., 2017a). In this study, we aimed to use inflammatory pain rats to determine whether the analgesic effect of galanin is accomplished by activating GALR2 and to explore the underlying signaling mechanism. Some studies have demonstrated that the subcutaneous injection of carrageenan into the plantar region of the hind paw of animals can induce local inflammation and pain (Metcalf et al., 2015; Yang et al., 2015; Zhang et al., 2017a). The responses to noxious stimuli were enhanced during the inflammatory process, and bilateral HWL to noxious thermal stimulation and HWT to mechanical stimulation decreased 3 or 4 h after the injection of carrageenan (Sun et al., 2003; Xiong et al., 2005; Yang et al., 2015). In this study, animal models were established through injecting carrageenan into the plantar of left hind paw of rats.

An early study showed that thermal noxious or intense chemical stimulation could induce pain perception by an ascending nociceptive modulation and that this effect depended on both opioid and dopamine links in the NAc (Gear et al., 1999). The infusion of N-acetylaspartylglutamate into the NAc significantly attenuated the pain induced by activation of sensory nerves through optical stimulation (Watanabe et al., 2018). These studies suggested that the NAc plays an important role in mediating the suppression of tonic or persistent pain. As early as 1992, Kordower et al. (1992) reported that galanin-immunoreactive fibers were seen within the NAc in monkey, therefore the potential role of galanin in NAc on pain modulation is worth investigation.

Galanin and GALRs expressed at the sites of pain mediation. Xu et al. (2012) demonstrated that after sciatic nerve-pinch injury, GALR1 expression was up-regulated in spinal dorsal horn, whereas GALR2 was also up-regulated in both dorsal root ganglion and spinal dorsal horn. Our previous study showed that the expressions of both galanin and GALR1 were up-regulated in the NAc of rats with neuropathic pain (Duan et al., 2015; Zhang et al., 2019), while other studies showed that administration of GALR1 agonist M617 to intracerebroventricular (Fu et al., 2011), central nucleus of amygdala (Li et al., 2012), NAc (Duan et al., 2015) could induce a significant analgesic effect in rats, implying that GALR1 mediates the galanin-induced antinociceptive effect in rats. In addition, our previous study showed 3 h after carrageenan injection that the expressions of both GALR1 and GALR2 were significantly upregulated in NAc (Yang et al., 2015). Zhang et al. (2017a) also reported that the level of GALR2 in the ACC was increased in rats with acute inflammation (Zhang et al., 2017a). Compared with GALR1 and GALR2, there were fewer studies about the role of GALR3 in pain manipulation. Lv et al. (2019) reported that central spexin, a natural ligand for GALR2/3, produced an antinociceptive effect by activating GALR3 in the acute inflammatory pain models (Lv et al., 2019).

Whether GALR2 plays an antinociceptive role in the NAc of inflammatory rats was not examined until now. In the present study, we aimed to study the analgesic effect of GALR2 activation on inflammatory pain, and the first result found that galanin-induced analgesic effect was weakened by the intra-NAc administration of GALR2 antagonist M871 in rats with inflammatory pain. Consistent with this result, other studies also found that the administration of exogenous GALR2 antagonist M871 to the periaqueductal gray (PAG; Zhang et al., 2015), ACC (Zhang et al., 2017a) attenuated the galanin-induced analgesic effects in rats. These results together suggested that galanin might have an analgesic effect on inflammatory pain which was mediated by GALR2. But in this study, we also found that the antinociception of galanin was blocked partially by GALR2 antagonist M871, implying that galanin in NAc has an analgesic effect on inflammatory pain by activating other GALRs. In this study, to further prove the role of GALR2 on inflammatory pain, the GALR2 agonist M1145 was injected into the NAc of rats 3 h after carrageenan injection, the results showed that M1145 dose-dependently increased bilateral HWL to thermal stimulation and HWT to mechanical stimulation, and the M1145-induced analgesic effect was blocked by the intra-NAc administration of GALR2 antagonist M871. These results further confirmed that the analgesic effect of galanin on inflammatory pain was achieved through activating GALR2, although the underlying signaling mechanism is unclear.

Considerable evidences have shown that Ca^2+^-mediated signaling pathways are important in nociception. A large amount of Ca^2+^ enters into the cell and then activates intracellular Ca^2+^-dependent protein kinases, including CaMKII and PKC (Lisman et al., 2002). Former studies have reported that p-PKC is upregulated in dorsal root ganglion (DRG) neurons of rats with inflammatory arthritis pain (Koda et al., 2016; Bai et al., 2019). Consistently, the data of our present study showed that the expressions of both p-PKC and p-CaMKII in the NAc were upregulated 3 and 4 h after carrageenan injection in rats, which indicated that PKC and CaMKII in NAc were involved in the modulation of inflammatory pain of rats.

GALR2 mainly couples to G_q/__11_-type G-protein, therefore activation of GALR2 leads to phospholipase C (PLC) activation (Wittau et al., 2000), and causes calcium mobilization. Therefore, we conjected that the analgesic effect of GALR2 activation on inflammatory pain was achieved through the Ca^2+^-mediated signal transduction pathways. Former studies showed that administration of the PKC inhibitor chelerythrine into cerebroventricular (Shi et al., 2011) or central nucleus of amygdala (Li et al., 2017) significantly inhibited galanin-induced analgesic effect in rats, while administration of the CaMKII inhibitor MAP into PAG also inhibited galanin-induced analgesic effect in rats (Zhang et al., 2015). Based on these studies, in this work, we focus on whether the PKC and/or CaMKII signaling pathways in the NAc underlay the GALR2 activation-induced analgesic effects in inflammatory pain rats. Interestingly, the results of this study showed that p-PKC and p-CaMKII in the NAc were downregulated after the administration of the GALR2 antagonist M871 in inflammatory pain rats, suggesting that PKC and CaMKII might be involved in the GALR2 activation-induced analgesic effect in the NAc of inflammatory pain rats. To further explore the underlying mechanism, in the present study, M1145 was injected into the NAc of rats with inflammatory pain, and the CaMKII inhibitor KN93 or PKC inhibitor GO6983 was injected into the NAc 5 min after the intra-NAc administration of M1145. The results showed that KN93 and GO6983 weakened the M1145-induced increases in the HWL and HWT of inflammatory pain rats. These results illustrated that GALR2 activation in the NAc had an analgesic effect on inflammatory pain via PKC and CaMKII signaling pathways in rats.

**FIGURE 8:**
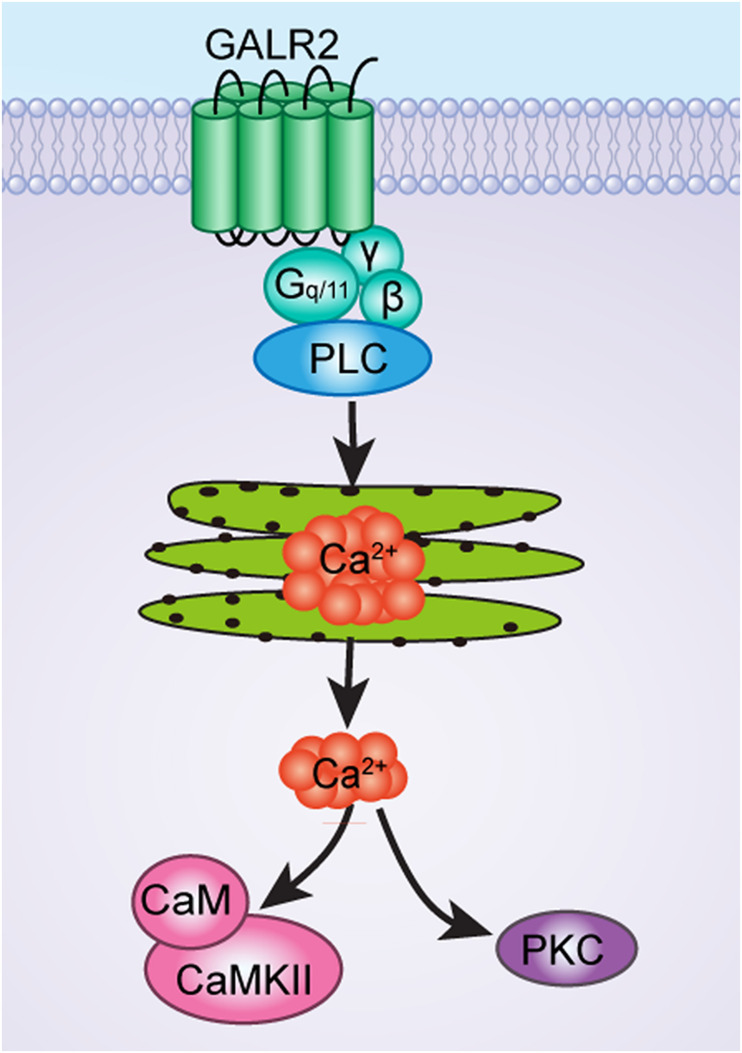
The schematic diagram of CaMKII and PKC signaling pathways of GALR2-induced analgesic effect in NAc of inflammatory pain rats.

As has been mentioned earlier, GALR2 couples to G-protein (G_q/__11_-type), which causes PLC activation and calcium mobilization, and may then lead to the recruitment of Ca^2+^-dependent PKC and CaMKII, as shown in Figure 8. Therefore, it is possible that after the injection of PKC and CaMKII inhibitors to block the PKC and CaMKII signaling pathways, GALR2-induced analgesic effect is then weakened.

Taken together, all the results of this study show that CaMKII and PKC are involved in inflammatory pain. GALR2 activation in the NAc results in an analgesic effect on inflammatory pain via the activation of PKC and CaMKII in rats. This finding implies that GALR2 agonists may be potent relievers of inflammatory pain.

## Data Availability Statement

The raw data supporting the conclusions of this article will be made available by the authors, without undue reservation.

## Ethics Statement

The animal study was reviewed and approved by the Animal Care and Use Committee at Kunming Medical University.

## Author Contributions

SX: study concept and design. ML, YL and SY: acquisition of data. SX and CL: analysis and interpretation of data. XZ and SX: drafting and revision of the manuscript. SX and XZ: funding. SX and CL: study supervision. All authors contributed to the article and approved the submitted version.

## Conflict of Interest

The authors declare that the research was conducted in the absence of any commercial or financial relationships that could be construed as a potential conflict of interest.
